# Cytomegalovirus-induced peroxynitrite promotes virus entry and contributes to pathogenesis in a murine model of infection

**DOI:** 10.1128/mbio.03152-23

**Published:** 2024-07-02

**Authors:** Pragati S. Amratia, Lauren E. Kerr-Jones, Lucy Chapman, Morgan Marsden, Mathew Clement, Richard J. Stanton, Ian R. Humphreys

**Affiliations:** 1 Division of Infection and Immunity/Systems Immunity University Research Institute, Cardiff University, Cardiff, United Kingdom; Griffith University - Gold Coast Campus, Griffith, Australia

**Keywords:** cytomegalovirus, inflammation, viral replication, oxygen radicals

## Abstract

There are no licensed vaccines for human cytomegalovirus (HCMV), and current antiviral drugs that target viral proteins are toxic and prone to resistance. Targeting host pathways essential for virus replication provides an alternate strategy that may reduce opportunities for drug resistance to occur. Oxidative stress is triggered by numerous viruses including HCMV. Peroxynitrite is a reactive nitrogen species that is formed during oxidative stress. Herein, we identified that HCMV rapidly induces the generation of intracellular peroxynitrite upon infection in a manner partially dependent upon xanthine oxidase generation. Peroxynitrite promoted HCMV infection in both cell-free and cell-associated infection systems in multiple cell types. Inhibiting peroxynitrite within the first 24 hours of infection prevented HCMV replication and peroxynitrite promoted cell entry and pp65 translocation into the host cell nuclei. Furthermore, using the murine cytomegalovirus model, we demonstrated that antagonizing peroxynitrite significantly reduces cytomegalovirus replication and pathogenesis *in vivo*. Overall, our study highlights a proviral role for peroxynitrite in CMV infection and implies that RNS and/or the mechanisms that induce their production could be targeted as a novel strategy to inhibit HCMV infection.

**IMPORTANCE:**

Human cytomegalovirus (HCMV) causes significant disease in individuals with impaired or immature immune systems, such as transplant patients and after congenital infection. Antiviral drugs that target the virus directly are toxic and are susceptible to antiviral drug resistance due to virus mutations. An alternate strategy is to target processes within host cells that are required by the virus for replication. Herein, we show that HCMV infection triggers a highly reactive molecule, peroxynitrite, during the initial stages of infection. Peroxynitrite was required for the initial entry of the virus into the cell and promotes virus replication in multiple cell types, suggesting a broad pro-viral function. Importantly, targeting peroxynitrite dramatically inhibited cytomegalovirus replication in cells in the laboratory and in mice, suggesting that therapeutic targeting of this molecule and/or the cellular functions it regulates could represent a novel strategy to inhibit HCMV infection.

## INTRODUCTION

The intracellular redox micro-environment has an important role in regulating many intracellular processes (1–4). Reactive oxygen species (ROS) production and elimination are balanced in cellular homeostasis, creating a cytostatic environment (1–4). At both low and high concentrations, different types of ROS can serve as signaling molecules, driving multiple pathways and metabolic functions (1–4). These interactions can determine the fate of the cell in response to stimuli, with beneficial or deleterious effects depending on the context (1–4). Transient oxidative stress is required for conducive physiological signaling and, in response to stimuli, activates multiple signaling cascades necessary for intra- and intercellular signaling (1–4). However, prolonged oxidative stress can cause detrimental damage to DNA and other macromolecules, ultimately leading to the activation of pathways that trigger cell death (1–4). Consequently, cells deploy multiple cellular antioxidant defense mechanisms that are essential to modulate and maintain ROS at physiological levels (1–4).

Several types of ROS can be generated including superoxide (O2•−), hydroxyls (HO•), peroxyl radicals (ROO•), and nitric oxide (NO•) (5). Nitric oxide (NO·) can generate reactive nitrogen species (RNS), following a reaction with other free oxygen-containing radicals (1, 5). For example, the reaction of nitric oxide with superoxide generates peroxynitrite (ONOO−) and occurs following the simultaneous production of superoxide and nitric oxide in close proximity (6, 7). Peroxynitrite is classified as either ROS or RNS as it is a powerful oxidizing and nitrating agent (6, 7). It is highly diffusible across cellular membranes and at low concentrations and can activate multiple signaling pathways leading to the production of proinflammatory cytokines (1, 7, 8). Peroxynitrite can also directly interact with DNA to induce single or double-stranded breaks, which are often reversible through the activation of DNA repair pathways (1, 7). However, continuous production of peroxynitrite induces cellular damage (1, 7). Therefore, at higher concentrations, the effects of peroxynitrite shift from physiological to pathological, causing irreversible oxidation/nitration of biological molecules (1, 7).

Although oxidative stress and subsequent signaling events can enhance antiviral immune responses (9, 10), several studies have demonstrated that ROS promotes the replication of a broad spectrum of viruses including human immunodeficiency virus-1 (HIV), herpesviruses, and influenza (11–13). Viruses can trigger oxidative stress *via* multiple mechanisms including the generation of mitochondrial ROS and activation of NADPH oxidase (NOX) enzymes (11–13). Virus replication benefits from ROS production in several ways, including *via* redox regulation of viral proteins and exploitation of ROS-induced host signaling pathways (11–13). Interestingly, peroxynitrite has been shown to exert both anti- and pro-viral effects during infection. Peroxynitrite directly impinges on coxsackievirus replication by inhibiting the entry of viral RNA (14). By contrast, treatment with peroxynitrite scavengers inhibited HIV-1 replication in both acutely and chronically infected macrophages (15). Here, lack of peroxynitrite biosynthesis affected the expression of HIV core protein, p24, and its precursor, p55 *in vitro* (15). However, the paucity of data regarding the impact of peroxynitrite in different viral infections and how it influences replication *in vivo* has limited our understanding of the role that peroxynitrite plays in viral pathogenesis.

The beta-herpesvirus human cytomegalovirus (HCMV) infection is an example of a viral pathogen that exploits ROS during infection. HCMV infection induces ROS production through the alteration of mitochondrial function including increasing the activity of the electron transport chain (16, 17). Significant intracellular superoxide production is observed during lytic HCMV infection *in vitro,* and HCMV-induced ROS is essential for the expression of transactivation of the major immediate promoter (MIEP), immediate-early viral protein IE72, and subsequent viral replication (18, 19). Antioxidants inhibit CMV immediate-early gene expression, viral replication, and CMV-induced activation of nuclear factor κB (NF-κB) (18, 19). Conversely, HCMV exerts control of intracellular ROS production to protect the host cell *via* induction of the antioxidant glutathione (20). Furthermore, to maintain gene silencing during latency, HCMV prevents ROS generation through the expression of b2.7-long non-coding RNA that quenches ROS and limits NF-κB expression (21).

Published data clearly demonstrate the importance of ROS in HCMV replication (12, 13, 17–20). However, given the broad biological functions of ROS (1–4), a better understanding of ROS-mediated mechanisms that facilitate virus replication, including identification of the molecules involved, may inform our understanding of virus replication and identify novel targets for antiviral therapeutic strategies (11, 12, 22). Despite the suggested importance of peroxynitrite in viral pathogenesis (1, 14, 15), the role that this ROS plays in HCMV infection is unknown. Thus, we investigated the role that peroxynitrite plays during CMV infection. We reveal that HCMV infection leads to the rapid generation of peroxynitrite during infection. Using multiple peroxynitrite antagonists, we demonstrate that peroxynitrite exhibits pro-viral activity during the initial stages of HCMV replication prior to viral entry into the nucleus and facilitates HCMV replication in cell-free and cell-to-cell infection settings. Moreover, using the murine cytomegalovirus (MCMV) model of infection, we found that administration of a peroxynitrite scavenger dramatically inhibits CMV replication and associated pathogenesis *in vivo*. Overall, these studies identify peroxynitrite and its generation as a putative target for antiviral therapeutic approaches.

## RESULTS

### Peroxynitrite is induced by HCMV infection and promotes viral replication *in vitro*

**Fig 1:**
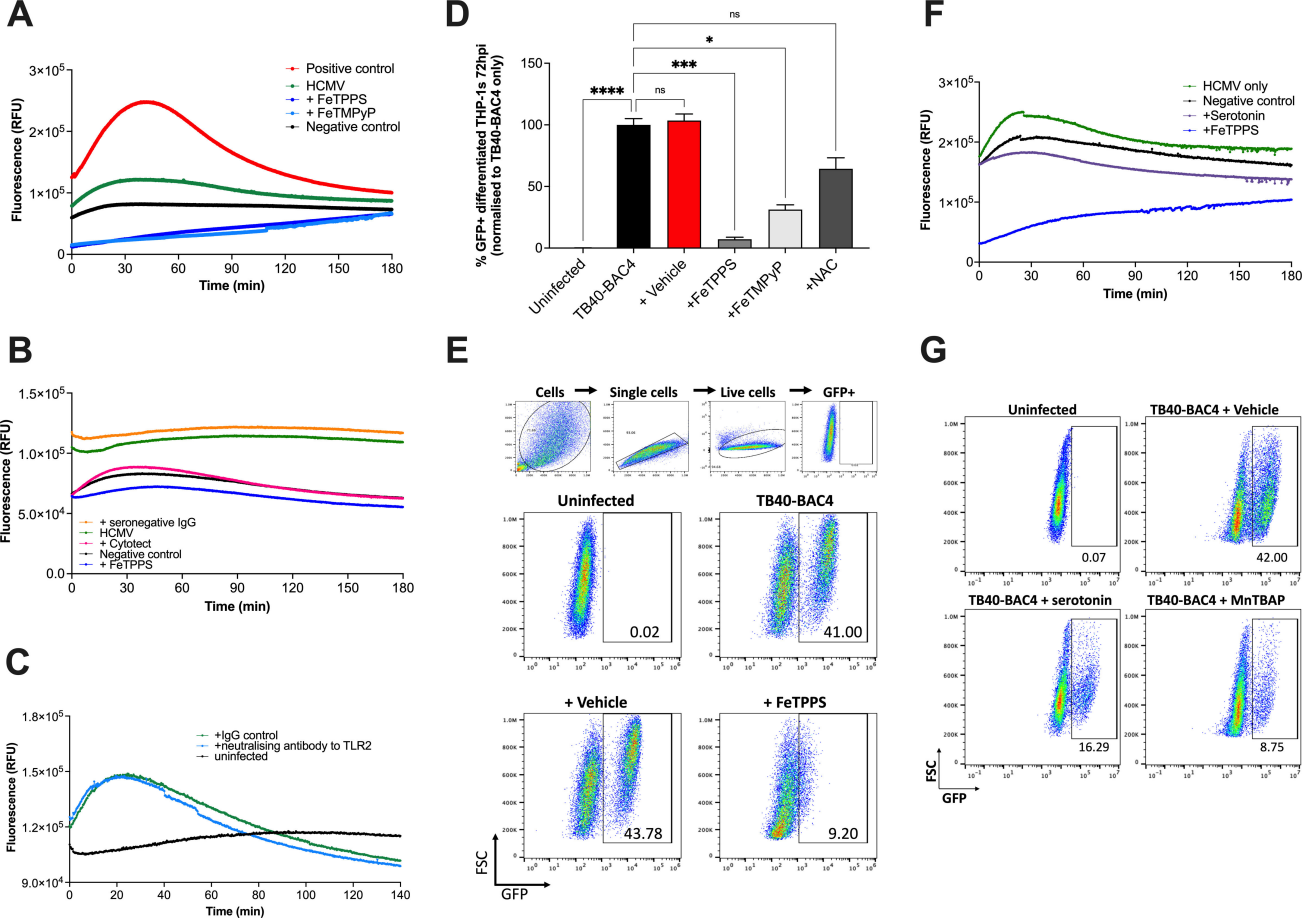
Peroxynitrite is essential for HCMV infection in PMA-differentiated THP1 cells *in vitro*. (**A**) Peroxynitrite production in PMA-differentiated THP-1 cells treated with peroxynitrite scavengers before cell-free TB40-BAC4 infection (MOI 25). (**B**) HCMV was pre-incubated with Cytotect or seronegative IgG (each at 40 µg/mL) before peroxynitrite measurement. (**C**) Cells were pre-treated with neutralizing antibody against TLR2 or IgG control (200 µg/mL) before the addition of HCMV and measurement of peroxynitrite generation. (**D**) Percentage of HCMV infected (GFP+) THP-1 cells 72 hpi, following pre-treatment with inhibitors (*n* = 4–7). Data plotted as mean ± SEM. Statistical analysis using the Kruskal-Wallis test with Dunn’s multiple comparisons (****P < 0.0001; ***P < 0.0005; *P < 0.01, and ns, not significant). The gating strategy and representative flow cytometry plots are shown in (**E**). Only live cells were selected for analysis. (**F**) Peroxynitrite production in differentiated THP-1 cells treated with serotonin before cell-free TB40-BAC4-GFP infection (MOI 25). (**G**) Representative flow cytometry plots of HCMV infected (GFP+) THP-1 cells pre-treated with serotonin, MnTBAP, or vehicle. (**A-B** & **E**) Fluorescence intensity was measured every 10 s for 180 min. Average fluorescence was plotted (*n* = 2). Cells were pre-treated with FeTPPS (25 µM), FeTMpYP (25 µM), NAC (10 mM), Serotonin (250–500μM), MnTBAP (50 µM), or vehicle. Data are representative of at least two separate experiments.

Cells of the myeloid lineage are important cellular host targets for HCMV. We first measured peroxynitrite generation during HCMV infection of myeloid cells, using the THP-1 cell line. THP-1s were differentiated with phorbol 12-myristate-13-acetate (PMA) into macrophage-like cells which permit lytic replication (23–25). Before infection with an HCMV strain expressing GFP from immediate early time points (TB40-BAC4-GFP), differentiated THP-1 cells were incubated with a fluorescent probe that binds intracellular peroxynitrite. This produces a fluorescent product and enables the detection of peroxynitrite generation in real time. Using this assay, we observed that HCMV rapidly induced significant peroxynitrite production in the first 10 minutes of infection as compared to uninfected controls (Fig. 1A). To ensure that peroxynitrite generation was HCMV specific, the cell-free virus was incubated with CytotectCP (clinical-grade CMV-specific hyperimmunoglobulin pooled from multiple donors with high anti-HCMV neutralizing titers (26, 27)) an hour prior to the assay. Cytotect treatment diminished HCMV induction of peroxynitrite, similar to FeTPPS-treated cells and negative controls (Fig. 1B). By contrast, HCMV incubated with immunoglobulins (IgG) from HCMV seronegative donors retained the ability to induce peroxynitrite production (Fig. 1B), indicating this process was driven by HCMV binding and/or infection. TLR2 signaling triggers the generation of inflammatory cytokines following binding of HCMV glycoproteins (28, 29). Antibody neutralization of TLR2 had a moderate but reproducible (observed in three separate experiments) impact on HCMV-induced peroxynitrite generation (Fig. 1C), suggesting that activation of TLR2 upon HCMV binding partially contributes to HCMV-induced peroxynitrite generation.

Next, we assessed whether peroxynitrite impacted HCMV infection. We treated differentiated THP-1 cells with peroxynitrite scavengers (FeTPPS or FeTMPyP), which we demonstrated strongly inhibited HCMV-induced peroxynitrite generation (Fig. 1A and B). As a comparator, some cells were treated with N-acetylcysteine (NAC), a hydrogen peroxide inhibitor previously demonstrated to inhibit HCMV replication in human foreskin fibroblast cells (30). Strikingly, pre-treatment of THP1 cells with FeTPPS or FeTMPyP reduced the proportion of infected cells 72 hours post-infection (hpi) by approximately 80% and 60%, respectively (Fig. 1D). A near-complete inhibition of HCMV infection was achieved with FeTPPS, as compared to vehicle control-treated cells (Fig. 1D and E). By contrast, the antiviral effect of NAC pre-treatment on HCMV infection was more subtle and did not reach statistical significance in our assay (Fig. 1D). Of note, the antiviral activity of FeTPPS and FeTMPyP was not accompanied by any impact on cell viability (<4% in all vehicle control-treated and FeTPPS/FeTMPyP-treated samples).

Furthermore, we also assessed whether a naturally occurring peroxynitrite scavenger, serotonin (31), which is structurally distinct from FeTPPS and FeTMPyP, could also inhibit HCMV replication. Although serotonin scavenging potential was less marked than that observed after pre-treatment with FeTPPS (Fig. 1F), we observed a twofold reduction of HCMV infection of differentiated THP1 cells following treatment (Fig. 1G). In addition, a synthetic manganese-based peroxynitrite decomposition catalyst (MnTBAP) had similar effects (Fig. 1G). Thus, overall, these data suggest that peroxynitrite facilitates HCMV infection.

### Peroxynitrite is required for efficient HCMV infection in multiple cell types

**Fig 2:**
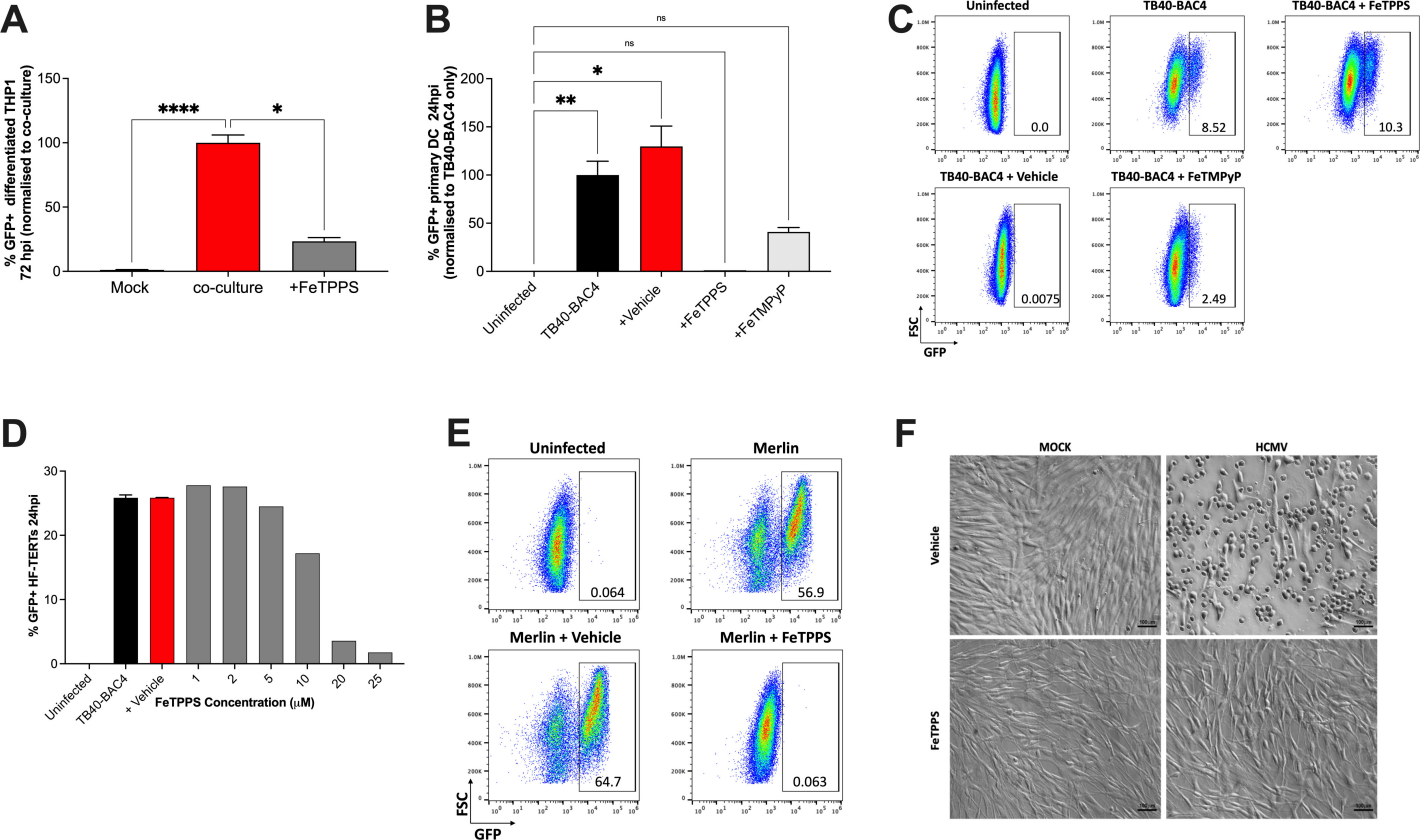
Peroxynitrite is essential for HCMV infection in multiple different cell types (**A**) Percentage of PMA-differentiated THP-1 cells infected by co-culture with Merlin-GFP-infected HF-TERTs (1:3 ratio) (*n* = 6–9). (**B-C**) The proportion of monocyte-derived dendritic cells infected with TB40-BAC4-GFP (MOI 25) after pre-treatment with peroxynitrite decomposition catalysts (each at 25 µM) (*n* = 2–4). Representative flow cytometry plots are shown in (**C**). (**D**) Dose-dependent effect of FeTPPS pre-treatment on HF-TERTs infected with TB40-BAC4-GFP (MOI 5) at 24 hpi. (**E**) Representative FACS plots of FeTPPS-treated HF-TERT at 24 hpi with Merlin-ΔUL128-GFP (MOI 5). (**F**) Transmission electron microscopy images of HF-TERTs pre-treated with FeTPPS or vehicle before infection with Merlin (MOI 5). Cell cultures were fixed at 24 hpi. Scale bar: 100 µm. (**A-B**) Data plotted as mean ± SEM. Statistical analysis using the Kruskal-Wallis test with Dunn’s multiple comparisons (****P < 0.0001; **P < 0.005; *, *P* < 0.01, and ns, not significant).

HCMV dissemination can occur *via* free virus particles (cell-free) or by direct cell-to-cell contact (26). Previous studies have shown that clinical HCMV isolates can efficiently spread through cell monolayers even though high titers of infectious cell-free viruses are not generated (26, 32). In animal models, virus dissemination through tissues is dependent on cell-to-cell spread (33) and virus *in vivo* is predominantly cell associated (26, 34, 35), as are recent clinical isolates. Thus, spread by direct cell-to-cell contact is likely critical *in vivo*, at least in part because it protects against neutralizing antibodies (26, 36). To investigate whether peroxynitrite is required for this process, we examined a HCMV strain (Merlin) that contains a genome matching the isolate derived from a patient and, therefore, spreads almost exclusively *via* cell-cell dissemination (26, 32, 35). Infected HF-TERTs were fluorescently labeled, and then incubated with PMA-differentiated THP-1 cells in the presence of FeTPPS. HCMV-infected THP-1 cells (unlabeled) were quantified 72 h later by flow cytometry. Notably, FeTPPS treatment significantly reduced contact-dependent HCMV infection of PMA-differentiated THP-1 cells (Fig. 2A), indicating that peroxynitrite is essential for both cell-free and cell-to-cell HCMV infection. Furthermore, these results suggest that the effect of peroxynitrite on HCMV replication was not virus-strain-specific.

Next, we examined whether peroxynitrite promoted HCMV replication in primary human cells as they retain most molecular and functional properties *ex vivo* and thus are more physiologically relevant models for studying viral infections (37). Human monocyte-derived dendritic cells were infected with TB40-BAC4-GFP in the presence of FeTPPS or FeTMPyP. Both peroxynitrite scavengers inhibited HCMV infection (Fig. 2B and C). We also determined whether peroxynitrite was required for lytic HCMV replication in non-hematopoietic cells. Pre-incubation of fibroblasts (HF-TERTs) with FeTPPS prior to HCMV TB40-BAC4-GFP infection reduced infection efficiency in a dose-dependent manner (Fig. 2D). Similarly, Merlin-GFP infection of HF-TERT cells was reduced to comparable levels as seen in uninfected control groups (Fig. 2E) with no impact on cell viability (Fig. 2F). Thus, these data suggest a broad requirement for peroxynitrite in lytic HCMV infection.

### Peroxynitrite promotes HCMV cell entry

**Fig 3:**
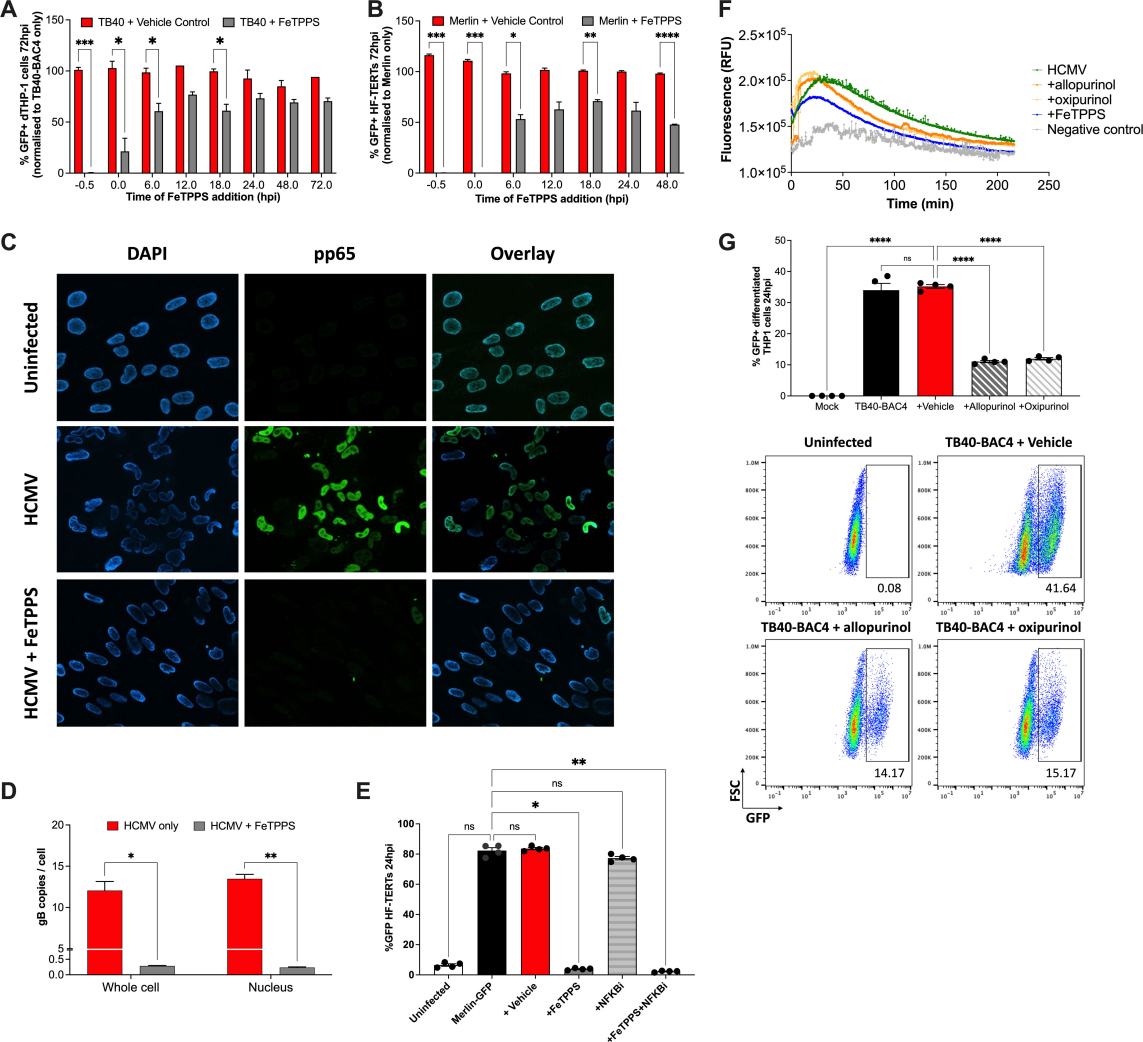
Peroxynitrite is essential during the initial stages of lytic HCMV infection. (**A**) Differentiated THP-1 cells and (**B**) HF-TERTs were infected with cell-free TB40-BAC4-GFP (MOI 25) and Merlin-ΔUL128-GFP (MOI 5), respectively, and treated with FeTPPS at different time points (*n* = 3–5). (**A-B**) All samples were treated with FeTPPS (25 µM) or vehicle at 6-hourly intervals and processed together at 72 hpi. The proportion of infected cells (GFP+) was measured by flow cytometry. Data plotted as mean ± SEM. Significance was determined by Sidak’s multiple comparisons tests, comparing the infected vehicle-control to its time-matched infected FeTPPS-treated sample (*P < 0.05; **P < 0.01, and ***P < 0.001). (**C**) Fluorescent microscopy images of cultures of fibroblasts that were pre-treated with FeTPPS (25 µM) or vehicle (H2O control) before cell-free infection with Merlin (MOI 5). Cells were stained for nuclei (DAPI, blue) and CMV-pp65 (CA003, green) at 24 hpi. All images were taken under ×40 magnification using an Axio Observer Z1 Zeiss microscope and are representative of two independent experiments. (**D**) HCMV genomes in HF-TERTs following cell-free infection with Merlin (MOI 5) in the presence or absence of FeTPPS (25 µM). HCMV genome levels were quantified by qPCR at 24 hpi and data were plotted as mean ± SEM (*n* = 3). (**E**) HF-TERTs were pre-treated with or without IKK16 and/or FeTPPS for 1 h and then infected with HCMV (MOI 5) (**F**) Peroxynitrite production by differentiated THP-1 cells was measured after pre-treatment with XO inhibitors, allopurinol, or oxipurinol (250 µM) prior to cell-free TB40-BAC4 infection. FeTPPS only was used as a control. (**G**) Differentiated THP-1 cells were pre-treated with XO inhibitors and infected with cell-free TB40-BAC4-GFP (MOI 25). Samples were processed at 24 hpi. Top: Data are shown as individual replicates, mean ± SEM. Bottom: Representative bivariant FACS plots. (D, E, & G) Statistical significance was determined by Dunnett’s multiple comparisons test (*P < 0.05; ****P < 0.0001, and ns, not significant).

We sought to investigate at which point in the virus replication cycle peroxynitrite was required by HCMV. Two HCMV strains TB40-BAC4-GFP and Merlin-GFP were used to directly infect PMA-differentiated THP-1 cells and HF-TERTs, respectively. FeTPPS was added at 6-h intervals following cell-free HCMV infection and quantified by flow cytometry at 72 hpi. The results revealed a time-dependent anti-HCMV activity of FeTPPS treatment in THP1-derived macrophages (Fig. 3A) and fibroblasts (Fig. 3B). The maximal anti-viral effect of FeTPPS occurred within the first 12 h in both cell types (Fig. 3A and B). The most profound effect on HCMV infection was achieved with inhibition of peroxynitrite prior to or at the onset of HCMV inoculation (≥99% or ≥79% inhibition, respectively) (Fig. 3A and B), implying that peroxynitrite was essential for facilitating cell entry and/or initiating viral replication.

To test whether peroxynitrite was required for HCMV cell entry, we assessed pp65 levels within the cell following FeTPPS treatment. HCMV pp65 is a major tegument protein that is delivered by incoming virions and translocates to the nucleus soon after virus entry (38). FeTPPS pre-treatment prevented nuclear translocation of CMV pp65 following cell-free infection (Fig. 3C), implying that virion entry and/or intracellular trafficking were inhibited. To differentiate these possibilities, we measured viral genome copy numbers at 24 hpi (prior to initiation of viral DNA synthesis (39)) in fibroblasts pre-treated with or without FeTPPS before cell-free infection with Merlin. FeTPPS treatment led to a >20-fold decrease in viral genome copies in both the whole cell and nuclei (Fig. 3D). NF-κB activation is important for the initiation of viral DNA synthesis during HCMV replication (18). Antagonizing NF-κB with the inhibitor IKK-16, which targets IκB kinase (IKK) and inhibits HCMV-induced inflammatory cytokine production (29), had little impact on HCMV infection at 24 hpi (Fig. 3E), suggesting that HCMV-induced peroxynitrite facilitates virus entry predominantly in an NF-κB-independent manner. These data collectively suggest a role for endogenous peroxynitrite in promoting HCMV cell entry at an early stage of the replication cycle.

Given that peroxynitrite was induced and exerted pro-viral activity in the initial stages of HCMV replication, we next investigated the source of ROS required for virus replication at this time. Peroxynitrite generation is spatially situated near sources of superoxide, which is shorter-lived, comparatively unstable, and not as diffusible across membranes as nitric oxide (7). Xanthine oxidase (XO) is a significant source of ROS and has been shown to play a crucial role in innate inflammatory signaling (40). Importantly, in vascular endothelial cells, XO is a source of early ROS production following HCMV infection (18). Thus, we inhibited XO with two different XO inhibitors (allopurinol and oxipurinol) prior to HCMV infection. Pre-treatment with either inhibitor antagonized CMV-induced peroxynitrite function (Fig. 3F) and significantly decreased HCMV infection in THP-1 cells 24 hpi (Fig. 3G), implying the importance of early XO activity in peroxynitrite production.

### Inhibition of peroxynitrite inhibits MCMV replication *in vitro* and *in vivo*

**Fig 4:**
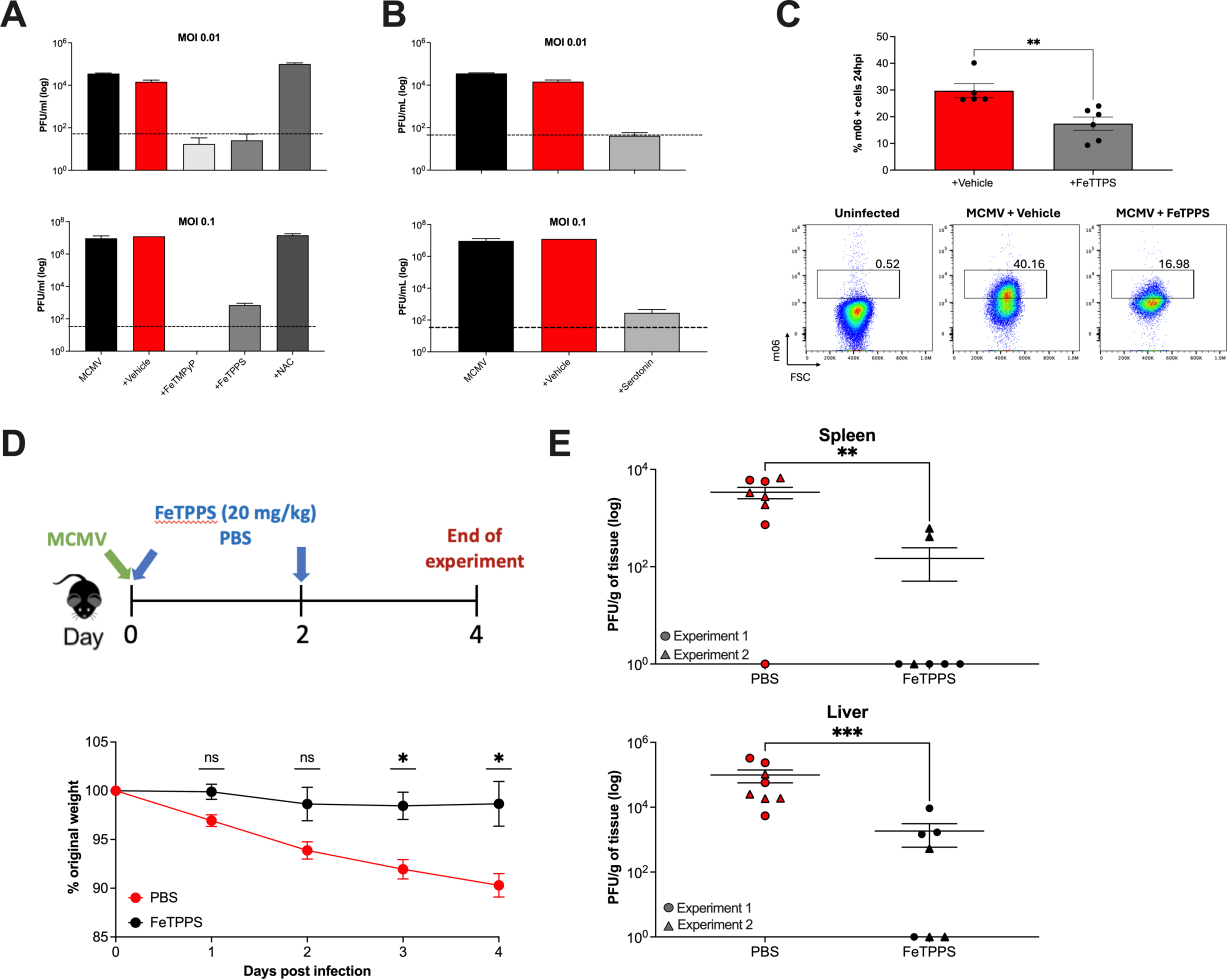
Peroxynitrite facilitates MCMV replication *in vitro* and *in vivo*. (**A-B**) Murine fibroblasts were infected with MCMV (MOI of 0.01 or 0.1) and treated with FeTMPyP (100 mM), FeTPPS (100 mM), NAC (10 mM) (**A**), or serotonin (500 mM) (**B**), with vehicle control. The supernatant was collected on day 4 after infection and MCMV concentration (PFU/mL) was quantified using a plaque assay. Data plotted as the average PFU/mL from 2 to 3 replicates. The limit of detection is shown as dotted lines. (**C**) Murine 3T3 fibroblasts were infected with MCMV (MOI 5) and MCMV m06 protein was detected at 24 hpi by flow cytometry. (**D**) Schematic diagram of the experimental procedure (top). C57BL/6 mice were infected with MCMV and were treated with FeTPPS (*n* = 3) or PBS (*n* = 4) at days 0 and 2pi. Percentage change in body weights over 4 days was plotted (bottom). (**E**) Viral load in spleen and liver samples of FeTPPS-treated and PBS-control mice after MCMV infection, calculated using plaque assay. Individual mice are shown and individual values from each mouse were used for statistical analysis. Data were pooled from two independent experiments (represented as circles and triangles) and plotted as mean ± SEM PFU/mg of tissue. Mann-Whitney U test was performed to determine statistical significance (*P < 0.05; **P < 0.005; ***P < 0.001).

To investigate whether peroxynitrite could be targeted to improve control of CMV replication *in vivo*, we studied the impact of FeTPPS in the murine cytomegalovirus (MCMV) model of infection. We first demonstrated that treatment with the peroxynitrite scavengers FeTPPS, FeTMPyP (Fig. 4A), and serotonin (Fig. 4B) potently inhibited MCMV replication in murine fibroblasts, leading to a ~ 2 log decrease in viral load detected in culture supernatant after 4 days following a low MOI infection. Furthermore, FeTPPS inhibited initial viral infection after high MOI infection, as determined by the detection of MCMV m06 protein following *in vitro* infection of fibroblasts (Fig. 4C). We next examined whether peroxynitrite inhibition reduced MCMV replication *in vivo*. MCMV-infected mice were treated with FeTPPS or PBS at the time of infection and 48 hpi (Fig. 4D). Over a 4-day experiment, FeTPPS treatment prevented weight loss, with treated mice maintaining their weight throughout (Fig. 4D). Reduced weight loss in FeTPPS-treated mice was accompanied by a significant reduction in viral load in both spleens and livers of MCMV-infected mice, eliminating detectable MCMV replication in some mice (Fig. 4E). Thus, these data highlight peroxynitrite as an important cellular factor that facilitates CMV replication *in vitro* and *in vivo*.

## DISCUSSION

Herein, we have identified the role of peroxynitrite, a potent oxidant and nitrating agent, in promoting CMV replication. HCMV rapidly induced peroxynitrite generation upon infection which was partially dependent upon the induction of xanthine oxidase. Treatment with peroxynitrite scavengers dramatically inhibited virus infection in both *in vitro* and *in vivo* models of CMV infection. Peroxynitrite inhibition prior to or at the onset of HCMV infection completely blocked viral infection in a range of susceptible cell types in cell-free and cell-to-cell infection systems, including monocyte-derived macrophages, dendritic cells, and fibroblasts. In the context of cell-free infection, peroxynitrite appeared to be essential for HCMV entry into the host cell, irrespective of whether this occurred *via* fusion at the plasma membrane (fibroblasts), or *via* the endosomal route (PMA-differentiated THP-1 cells).

The reaction of nitric oxide with superoxide to generate peroxynitrite occurs quickly when both superoxide and nitric oxide are present within close proximity (6, 7). In the context of HCMV, our data reveal that this reaction occurs within the first minutes of infection and requires virus binding to the cell. This observation is in accordance with the induction of ROS within the first few minutes of HCMV binding to smooth muscle cells (18), consistent with the idea that HCMV induces the production of superoxide, which, in turn, reacts with nitric oxide to form peroxynitrite. In smooth muscle cells (18), XO is implicated as a major source of ROS induced in the initial stages of infection, and we showed that XO inhibition both antagonizes the production of peroxytnitrite and inhibits HCMV replication in THP1 cells. In THP1 cells, XO activity is triggered upon activation of TLRs including TLR2 (41). HCMV induces TLR2 activation and cytokine production following the binding of its glycoproteins (28, 29). We also observed that TLR2 partially contributed to but was not entirely responsible for, CMV-induced peroxynitrite generation that facilitated virus entry. Thus, our data suggest that initial induction of XO upon HCMV infection contributes to peroxynitrite production, and activation of TLR2 may contribute to this process.

How peroxynitrite promotes HCMV infection is not well understood. Previous reports have shown that virus-induced ROS is required for HCMV replication by activating NF-κB, which, in turn, binds to and activates the major immediate early promoter (MIEP) and subsequent viral gene expression (18). At certain concentrations, peroxynitrite can also activate NF-κB (1, 8), implying that peroxynitrite may also be capable of promoting HCMV replication *via* similar mechanisms. However, inhibiting NF-κB had no impact on HCMV infection in our assays at time points at which peroxynitrite facilitated virus infection. Moreover, our data suggest that peroxynitrite impacts virus infection at the point of cell entry and/or intracellular trafficking. In the context of cell entry, HCMV entry into a broad range of permissive cells is coordinated by interaction between virally encoded glycoproteins and specific cell surface receptors (42, 43). HCMV glycoprotein gB and the trimeric complex gH/gL/gO interact with cellular integrins (42, 44) and receptor tyrosine kinases (RTKs) (42, 45), including platelet-derived growth factor receptor alpha (PDGFRα) (46–48) as well as epidermal growth factor receptor (EGFR) (49, 50). RTKs promote cell entry, triggering the activation of multiple signaling cascades, including ERK, MAPK, and PI3K signaling pathways which, in turn, facilitate processes such as localizing virus into trafficking pathways and nuclear translocation of viral DNA (42, 50). Peroxynitrite can post-translationally modify redox-sensitive groups (such as cysteine, methionine, and tyrosine residues) in these proteins, altering their structure and function (1, 7, 8). Thus, peroxynitrite induction of phosphorylation of tyrosine residues has the potential to activate RTKs to promote CMV cell entry.

HCMV causes a substantial disease burden in the immunologically immature following congenital infection and in the immune-compromised (51, 52). One cohort of these latter clinical grouping is solid organ transplant recipients where HCMV causes morbidity, acute graft rejection, and in some instances, mortality in patients (51, 52). In the context of kidney transplant recipients (KTRs), HCMV viremia and clinically significant infection is most common in HCMV seronegative individuals who receive kidneys from HCMV seropositive donors (D + R-) (51, 52). Interestingly, organ ischemia and reperfusion can lead to ROS production and oxidative stress (53). As recently highlighted by Perera and Sinclair (21), this process may be conducive to the induction of HCMV reactivation from latency. Our data imply that oxidative stress within the transplanted organ may also facilitate subsequent virus propagation through the formation of peroxynitrite. Thus, pharmacological agents that selectively target HCMV-induced peroxynitrite may represent a novel strategy for restricting HCMV pathogenesis in immune-suppressed graft recipients.

## MATERIALS AND METHODS

### Compounds

The peroxynitrite decomposition catalysts Fe(III)5,10,15,20-tetrakis(4-sulfonatophenyl)porphyrinato chloride (FeTPPS) and [[4,4′,4′′,4′′′-(21H,23H-porphine-5,10,15,20-tetrayl-κN^21^,κN^22^,κN23,κN^24^)tetrakis[1-methylpyridiniumato]](2-)]-Iron(5+), pentachloride (FeTMPyP), Mn(III)tetrakis(4-benzoic acid) porphyrin, monochloride (MnTBAP), serotonin hydrochloride, allopurinol, and oxipurinol were purchased from Cayman Chemicals. N-Acetyl-L-cysteine (NAC), an inhibitor of ROS, was obtained from Sigma-Aldrich. All compounds were freshly reconstituted in UltraPure DNase/RNase-Free Distilled Water (Invitrogen) or DMSO (Sigma-Aldrich) as per the manufacturers’ instructions on the day of the experiment. Anti-CMV IgG CytotectCP was purchased from Biotest and seronegative IgG was taken from seronegative donors (both used at 40 µg/mL).

### HCMV strains

Unless specified, HCMV strains expressing an enhanced GFP cassette linked to UL36 with a P2A self-cleaving peptide were used (35, 54). For cell-free infections, we used the Merlin HCMV strain with a single point mutation in the UL128 locus to enhance replication in fibroblasts (Merlin-ΔUL128-GFP) (55) or TB40-BAC4-GFP HCMV strain (56). HCMV were propagated in HF-TERTs as previously described (35). Supernatants from infected cells were harvested when 100% cytopathic effect (CPE) was observed, and then residual debris was removed through low-speed centrifugation. The cell-free virus was pelleted and viral titer was determined by plaque assay in HFFFs (57). Plaques were visualized using a Zeiss Axio Observer Z1 microscope. If small plaques were observed at day 14, cultures were incubated under a semi-solid overlay for further 7 days and recounted. For the assessment of cell-cell spread, we used an HCMV Merlin variant that had been repaired for UL128 and contains an eGFP cassette linked to UL36 with a P2A self-cleaving peptide (Merlin-GFP) (35). Since the presence of wild-type UL128 impairs the release of the cell-free virus, the UL128 locus was placed under conditional expression, enabling high titer cell-free propagation in HF-TERTs expressing tetracycline repressor (TET_R_) (35, 55). UL128 expression (and thus cell-associated growth) was restored in cells lacking TET_R_, enabling cell-cell spread to be assessed by infecting HF-TERTS, and then co-culturing with target cells (26).

### Cells

Human fetal foreskin fibroblasts (HFFFs), HFFFs immortalized with human telomerase reverse transcriptase (HF-TERTs) (58), and THP-1 cells (ATCC, TIB-202) were used in HCMV infection studies. THP-1 cells were differentiated into macrophage-like cells by culturing in a regular growth medium supplemented with 100 ng/mL PMA for 48 h (59). Differentiation was assessed by flow cytometry by measuring the expression of CD14 and CD11b (23). HCMV infections were carried out the following day, after overnight incubation in PMA-free media.

To generate monocyte-derived human dendritic cells, peripheral blood was obtained from an apheresis cone supplied by the Welsh Blood Service. CD14^+^ monocytes were isolated with magnetic-activated cell sorting (MACS) using CD14+ microbeads and LS columns (Miltenyi). The purity of the CD14+ sample was assessed by flow cytometry. Purified CD14^+^ monocytes were seeded into untreated cell culture plates, RPMI was supplemented with 10% FCS, IL-4 (100 ng/mL, Peprotech), GM-CSF (100 ng/mL, Peprotech), and β-Mercaptoethanol (50 nM, Gibco). Media and supplement changes were carried out every three days, excluding β-Mercaptoethanol which was only added on the day of isolation. The DCs were phenotyped 6 days post-purification by flow cytometry.

### HCMV infection assays

For cell-free infection, 1 × 10^6^ target cells were seeded in a T25 tissue culture flask in serum-free DMEM media. The following day, the cells were infected with HCMV at MOI 5 (unless stated otherwise to achieve comparable levels of infection in non-fibroblast) and incubated on a rocking platform. The inoculum was then removed and replaced with fresh media and cells prior to the assessment of GFP expression by flow cytometry. For co-culture infection, 1 × 106 HF-TERTs were seeded in a T25 tissue culture flask infected at MOI 5 and incubated on a rocking platform. After 72 h, infected HF-TERTs were stained with CellTrace Far Red DDAO-SE dye (Invitrogen), trypsinized, and resuspended in complete growth media prior to overlaying on 1 × 105 target cells. DDAO staining enabled HF-TERTs (DDAO+) to be distinguished from target cells (DDAO−). DDAO-stained uninfected HF-TERTs were used as control.

### Treatment with inhibitors

Cells were seeded in either a 24-well plate or a 25 cm^2^ tissue culture (T25) flask (Thermo Fisher Scientific) one day prior to assays. On the day of the assay, cells were treated with inhibitors. Prior to HCMV infection, inhibitors were removed, and cells were washed in PBS to ensure the virus was not in direct contact with scavengers. Cells were infected with HCMV at the required MOI and incubated on a rocking platform. After the inoculum was removed, cells were maintained in media containing inhibitors/controls throughout the assay. In some experiments, FeTPPS was added to media at different time points before and after HCMV infection. For assays targeting NF-κB or TLR2 signaling, IKK-16 (NF-κB inhibitor; IKK inhibitor VII, 1 nM; Cambridge Bioscience) or neutralizing antibodies to TLR2 (200 µg/mL; Invivogen) were added 1  h before the addition of HCMV (29).

### Measurement of intracellular peroxynitrite

The concentration of intracellular peroxynitrite was measured using a cell-based peroxynitrite assay kit (ab233470, Abcam). Samples were prepared as per the manufacturer’s protocol and were co-incubated with Peroxynitrite Sensor Green. The fluorescent signal was monitored after the addition of HCMV every 10–15 s for 3 h, by a fluorescence microplate reader (CLARIOstar, BMG Labtech, Germany). Uninfected cells stimulated with or without FCS were used as positive and negative controls, respectively.

### Flow cytometry

Cells were stained with Zombie Aqua Fixable (BioLegend) prior to antibody staining as per the manufacturer’s protocol and fixation with 4% paraformaldehyde. THP-1 cells were stained with anti-CD11b-PE/Cy7 (M1, BD Pharmingen) to confirm macrophage-like morphology. HCMV-infected cells were identified by GFP^+^ cells in the Zombie Aqua-negative population. Monocyte purity was assessed using anti-CD14-V500 (MφP9, BioLegend) or anti-CD14-FITC (M5-E2, BioLegend). DC purities were assessed using anti-CD14-PECy7 (eBioscience DX), CD1a-FITC (HI149, BD Pharmingen), and DC-SIGN-PE (DCN46, BD Pharmingen). Cells that were CD14-low, DC-SIGN-high, and CD1a-high were considered DCs. Unless stated otherwise, all data were acquired using Attune NxT Flow Cytometer (Thermo Fischer) and analyzed using FlowJo software (TreeStar).

### Detection of HCMV genome

Total DNA from cells was isolated using DNeasy Blood & Tissue kit (QIAGEN) as per the manufacturer’s instructions. DNA concentration was measured using an NG1000 NanoDrop spectrometer (Thermo Fisher Scientific) and was diluted in Ambion diethylpyrocarbonate (DEPC)-treated water (Thermo Fisher) such that the total amount of sample DNA was 100 ng per reaction. Each qPCR contained 100 ng DNA, 500 nM of each primer, 1 × iTaqTM Universal SYBR Green Supermix (Bio-Rad), and adjusted to a total reaction volume of 20 µL. The samples were prepared in triplicates and analyzed using Quant StudioTM3 Real-Time PCR System (Applied Biosystems). The PCR program started with reverse transcription at 50°C for 2 min and initial denaturation at 95°C for 10 min, followed by 40 cycles of denaturation at 95°C for 15 s and annealing/extension at 60°C for 1 min. Viral gene expression was quantified using primers gB-Forward (CTGCGTGATATGAACGTGAAGG) and gB-Reverse (ACTGCACGTACGAGCTGTTGG). The primers were designed to amplify the HCMV gB (UL55) gene. Expression of GAPDH (internal control) was amplified using primers GADPH-Forward (CCTCTGACTTCAACAGCGACAC) and GAPDH-Reverse (TGTCATACCAGGAAATGAGCTTGA). Serial dilutions of plasmids containing both genes and DNA extracted from uninfected HF-TERTs were used to generate a standard curve.

### Detection of pp65 HCMV antigen by immunofluorescence

HF-TERTs were seeded in ibidi μ-96-well black plates, and were pre-treated with FeTPPS or vehicle control 0.5 h prior to HCMV infection. Samples were kept in FeTPPS or vehicle control throughout the assay. After 24 h, cells were washed, fixed in 4% PFA, washed, and resuspended in 0.5% NP-40 solution prior to staining with anti-CMV pp65 antibody (CA003, Virusys). Infected cells were visualized with Alexa Fluor-488 (AF488) F (ab′)2-goat anti-mouse IgG (H + L) cross-adsorbed secondary antibody (A-11017, Invitrogen) with a DAPI nuclear stain. Samples were mounted (Dabco mounting solution, Sigma-Aldrich) prior to imaging. An Axio Observer Z1 (Zeiss) was used for fluorescence imaging. Nuclear dye DAPI (461 nm wavelength) and AF488-labeling (488 nm wavelength) were detected using blue and green fluorescence imaging channels, respectively. Zen2 software (Zeiss) was used to adjust exposure such that the background levels in uninfected samples were minimal. Images were captured independently for every channel and then merged to create a multi-channel image. A lens of ×40 with oil (Immersol 518F, Zeiss) was used.

### Mice, viral infections, and treatments

All *in vivo* studies were conducted under the UK Home Office-approved Project License (PPL No. P7867DADD) and in accordance with Home Office regulations. C57BL/6 mice (Charles River) were housed in pathogen-free scantainer cabinets at the Home Office designated animal research facility located at Heath Park Campus, Cardiff University. Mice were injected intraperitoneally (i.p.) with salivary gland-derived Smith strain MCMV (5 × 10^4^ PFU) generated as previously described (60). Mice were injected intraperitoneally with FeTPPS (25 mg/kg) or PBS at 0 and 2 dpi. After 4 days, the virus in homogenized tissues was titered on murine fibroblasts; NIH-3T3 cells (ATCC, CRL-1658). Mean values from technical replicates (triplicates) were used for analysis. High MOI MCMV infection was quantified as previously described in Stacey et al. (60) Briefly, cells were first infected with MCMV (MOI 5) and after 24 h, stained with Zombie Aqua dye, prior to fixation, permeabilization, and intracellular staining with anti-m06 antibody (CapRi) conjugated with allophycocyanin (APC) (Innova Biosciences) (60).

### Statistical analysis

Statistical significance was performed using PRISM9 GraphPad Software. Unless otherwise stated, the mean ± SEM is reported. The Mann-Whitney U test was used for paired analysis. The Kruskal-Wallis test with Dunn’s multiple comparisons was performed to compare three or more unmatched groups. When comparing several treatments to a single control, Dunnett’s comparison test was used. A mixed-effects model with Sidak multiple comparisons tests was performed to compare the infected vehicle to its time-matched infected FeTPPS-treated sample. For all tests performed, a *P*-value of ≤0.05 was considered to be significant. *P* values are reported as follows: non-significant (ns) >0.05; *≤0.05; **≤0.01; ***≤0.001 and ****≤0.0001.
